# A Serious Misapprehension

**Published:** 1912-03-02

**Authors:** Thomas Hayes

**Affiliations:** Clerk to the Governors. St. Bartholomew's Hospital, London, E.C.


*To the Editor of* THE HOSPITAL.

### A SERIOUS MISAPPREHENSION.

*To the Editor of* THE HOSPITAL.

SIR,—Considerable inconvenience has been caused by a statement which appeared in the Press on the 22nd inst. implying that St. Bartholomew's Hospital is offering treatment "for a weekly payment of £2."

I wish to say that no such offer has been made, nor has there at any time been a suggestion of making an offer of the kind.

The statement is, therefore, entirely without foundation so far as this hospital is concerned, and I can only assume it was made under a misapprehension.—I am, Sir, your obedient servant,

THOMAS HAYES,

Clerk to the Governors.

St. Bartholomew's Hospital, London, E.C.,
February 27.

